# Different risk and protective factors predict change of planning ability in middle versus older age

**DOI:** 10.1038/s41598-024-76784-1

**Published:** 2024-10-25

**Authors:** Josef M. Unterrainer, Julia Petersen, Peter Schmidt, Mareike Ernst, Markus A. Wirtz, Anna C. Reinwarth, Felix Wicke, Jasmin Ghaemi Kerahrodi, Matthias Michal, Thomas Münzel, Jochem König, Karl J. Lackner, Norbert Pfeiffer, Oliver Tüscher, Peter R. Galle, Manfred Beutel, Philipp S. Wild

**Affiliations:** 1 Institute of Medical Psychology and Medical Sociology, Faculty of Medicine, University of Freiburg, Hebelstraße 29, Freiburg, 79104 Germany; 2 Department of Psychosomatic Medicine and Psychotherapy, University Medical Center of the Johannes Gutenberg University Mainz, Mainz, Germany; 3 Department of Political Science and the Centre for International Development and Environment (ZEU), University of Giessen, Giessen, Germany; 4 Department of Clinical Psychology, Psychotherapy and Psychoanalysis, Institute of Psychology, University of Klagenfurt, Klagenfurt am Wörthersee, Austria; 5 Research Methods in the Health Sciences, University of Education Freiburg, Freiburg, Germany; 6 Department of Psychiatry and Psychotherapy, University Medical Center of the Johannes Gutenberg University Mainz, Mainz, Germany; 7 Partner Site RhineMain, German Center for Cardiovascular Research (DZHK), Mainz, Germany; 8 Division of Pediatric Epidemiology, Institute of Medical Biostatistics, Epidemiology and Informatics, University Medical Center of the Johannes Gutenberg-University Mainz, Mainz, Germany; 9 Institute of Clinical Chemistry and Laboratory Medicine, University Medical Center of the Johannes Gutenberg-University Mainz, Mainz, Germany; 10 Department of Ophthalmology, University Medical Center of the Johannes Gutenberg-University Mainz, Mainz, Germany; 11 Leibniz Institute for Resilience Research (LIR) Mainz, Mainz, Germany; 12 Department of Internal Medicine I, University Medical Center of the Johannes Gutenberg-University, Mainz, Germany; 13 Preventive Cardiology and Preventive Medicine, Center for Cardiology, University Medical Center of the Johannes Gutenberg-University Mainz, Mainz, Germany; 14 Center for Thrombosis and Hemostasis, University Medical Center of the Johannes Gutenberg- University Mainz, Mainz, Germany; 15 Center for Translational Vascular Biology (CTVB), University Medical Center of the Johannes Gutenberg-University Mainz, Mainz, Germany; 16 Institute of Molecular Biology (IMB) Mainz, Mainz, Germany

**Keywords:** Aging, Planning ability, Cognitive decline, Protective factors, Risk factors, Prospective cohort study, Neuroscience, Psychology, Medical research

## Abstract

Age-related cognitive decline has become an increasingly relevant public health issue. However, risk and protective factors of cognitive decline have yet to be investigated prospectively taking into account genetic, lifestyle, physical and mental health factors. Population-based data from middle-aged (40 to 59 years; *N* = 2,764) and older individuals (60 to 80 years; *N* = 1,254) were drawn from a prospective community cohort study using the Tower of London (TOL) planning task. Assessments were repeated at a 5-year interval to investigate age-related changes in planning performance and to determine the impact of risk and protective factors. Planning performance improved in middle-aged, but declined in older participants over 5 years. SNPs affecting the dopamine system (COMT, DRD2) and APOE polymorphisms differentially predicted cognitive performance in older vs. middle-aged individuals. For older individuals, high alcohol consumption, antidepressant medication and living without a partner had additional negative predictive power on cognition. In contrast, undiagnosed hypertension, no obstructive lung disease, and fewer years of education predicted cognitive decline in the middle-aged group. The results inform screening for individuals particularly vulnerable to cognitive decline and interventions (e.g., focusing on lifestyle factors) to help maintain cognitive performance into old age.

**Supplementary Information:**

The online version contains supplementary material available at 10.1038/s41598-024-76784-1.

## Introduction

The WHO has recently highlighted the diversity of aging trajectories and called for a better characterization of healthy aging^1^. Given this background of substantial heterogeneity, identifying risk and protective factors has become an indispensable step towards subsequent interventions aiming at delay or mitigation of cognitive decline. Traumatic brain injury, mid-life obesity, mid-life hypertension, smoking, diabetes, history of depression, sleep disturbances, and hyperlipidemia have been identified as risk factors. In contrast, years of education, physical activity, Mediterranean diet, cognitive training, moderate alcohol consumption, and social engagement reduce the risk for cognitive decline and dementia^2–4^. Factors that reflect somatic and psychological well-being as well as lifestyle could be subject to modification.

The average trajectory of cognitive performance from mid-adulthood to old age has been documented by using large samples (e.g^5^). Typically, comparatively lower test performance in memory, reasoning and perceptual speed becomes evident in cross-sectional studies from the third or fourth decade of life onwards (e.g^6^).

Notably, in longitudinal studies, another finding emerged: at their second visit, participants in mid-adulthood typically profit from prior cognitive test experience, even if the initial assessment was several years ago, as evidenced by moderately increased test performance. In contrast, from the seventh decade of life, at re-testing, lower performance was observed in memory and speed^7^. Salthouse^8^ concluded that older participants may be unable to generate a long-lasting benefit from the initial testing session. Hence, it may be a suitable approach to compare the predictive values of potential risk and protective factors in persons older vs. younger than 60 years to identify age-related predictors of cognitive performance.

Regarding the investigated cognitive domains, remarkably, executive functions have not yet been the focus of large longitudinal studies. This is surprising, since executive functions are strongly affected in “healthy” aging^9^. Executive functions such as inhibition, flexibility or planning underlie the organization and cognitively controlled pursuit of goal-directed behavior that is essential to mastering everyday life^10^. The consequences of their decline for an independent pursuit of life are pervasive^11,12^. A prototypical example of the controlled, multi-step, goal-directed organization of behavior is planning^13^. Using cross-sectional data, Unterrainer and colleagues^14^ found continuously lower planning performance in older participants from 40 to 80 years by the psychometrically validated version of the Tower of London task (TOL)^15^. This may render the TOL a suitable test to gauge age-related cognitive decline longitudinally.

The TOL depends on prefrontal cortex^16,17^ and on dopamine signaling via frontostriatal circuitries^18^. Both substantially change in aging (e.g^19–24^), suggesting the TOL as a promising measure for testing genetic influence on cognitive functioning.

Recent findings on the genetic predisposition of cognition have concluded that genetic background becomes increasingly important at older age (e.g^25^), a phenomenon termed “aging-related magnification” by Lindenberger and colleagues^26^. This may contribute to the increased interindividual variability of cognitive aging. Studies testing this hypothesis have mostly compared the effects of single-nucleotide polymorphisms (SNPs) on cognition between young adults and old participants. The most frequently studied candidate SNPs are polymorphisms of the dopamine system (COMT [rs4680 and rs4818], DRD1, DRD2, DBH) or the APOE, BDNF, and KIBRA genes (for reviews, see^27,28^. Several studies have tested executive functioning (e.g^29–31^), combinations of SNPs^32–35^, or additional factors such as hypertension or lifestyle^35,36^. However, to date, there is no population-based study that included all abovementioned SNPs and relevant risk and protective factors to predict age-related change in planning performance.

In summary, a multitude of risk and protective factors for cognitive aging have been reported. Trajectories of cognitive aging have been outlined, but there is still a need of studies focusing on executive functions, as these are essential to maintaining an independent living. Moreover, an increasing genetic influence can be expected in ageing^25^.

Here, we examined planning performance and its changes as assessed with the Tower of London - Freiburg Version (TOL-F) over a five-year interval in a community-based sample aged 40 to 80 years. Using structural equation modelling, a broad set of risk and protective factors were explored as predictors of cognitive change. More specifically, we included different physical and mental health measures, medication, lifestyle, acute, previous and chronic diseases, social support and socio-demographic factors and relevant SNPs to predict longitudinal change in planning performance. Analyses were stratified by age at T1 (</≥ 60 years), to identify age-dependent predictors of cognitive change.

## Methods

### Sample

The original Gutenberg Health Study (GHS) sample was drawn randomly from the local population register of the city Mainz (Germany) and the district Mainz-Bingen, stratified 1:1 for gender and residence and in equal strata for decades of age^37^. The inclusion criterion for the baseline examination was age 35 to 74 years. Exclusion criteria were insufficient knowledge of the German language and psychological or physical impairment prohibitive to participation in tests and interviews. Participants were examined for the first time between 2007 and 2012 in a standardized five-hour study center visit. The Tower of London was performed by 6,978 participants in the second run of the study between June 2012 and December 2015. Data of this study are based on the five-year (T1 in this study) and ten-year follow-up (T2) visits, when the TOL was used.

4,018 (*N* = 1,890 women, *N* = 2,128 men; *N* = 2,764 under the age of 60 at T1, *N* = 1,254 aged 60 or older at T1) participants who completed the five-year and ten-year follow-up and had no missing data points were included. 26.6% of participants who did the Tower of London at time 1 were no longer present at the second measurement. Reasons for drop-out were unwillingness to be followed up, inaccessibility, death and moving away from the study area.

The local ethics committee of the Medical Association Rhineland-Palatinate (Landesärztekammer Rheinland-Pfalz; ethic votes numbers 837.020.07 and 837.394.17) approved the GHS. All participants provided written informed consent. The GHS study’s review board approved the study protocol, which conforms to the principles outlined in the Declaration of Helsinki.

### Data collection and assessments

During their five-hour visit, participants filled out self-report questionnaires, including standardized psychometric measures. Computer-assisted interviews, anthropometric, and routine laboratory assessments were conducted in a standardized manner to assess cardiovascular risk factors, disease history (physician-diagnosed diseases), and humoral biomarkers. Medication was registered on site by scanning the bar codes of original packages of drugs taken by participants. Active ingredients in antidepressant, anxiolytic, antidiabetic and antihypertensive medication were recorded using ATC codes.

An overview of the assessed parameters is given in Table 1. More detailed information on the used instruments is provided in the Supplemental Information under “Data collection and assessments”.

**Table 1:** Overview of the assessed parameters and used instruments.

Overall Dimension	Parameter	Assessed by
*Cognition*	Planning ability	Tower of London – Freiburg Version (TOL-F): overall planning accuracy
*Socioeconomic factors and social support*	Sex/gender, age, partnership Level of education	Self-report Self-report (years)
	Social Support	Brief Social Support Scale (BS-6;^38^).
*Mental distress measures*	Loneliness Anxiety symptoms Depression symptoms Panic disorder Current mental state Current antidepressant and/or anxiolytic medication	Self-rating: “I am frequently alone/have few contacts”^39,40^. GAD-2^41^. Patient Health Questionnaire’s (PHQ) depression module^42^. Brief PHQ panic module^43^. Self-rating, range from 1 = very good, 2 = good, 3 = less good to 4 = bad Self-report (yes / no)
*Physical health and health behavior*	Cancer, cardiovascular disease, obstructive pulmonary disease (chronic OPD (COPD) and/or asthma), autoimmune diseases, chronic liver disease, chronic kidney disease, and infection last week	Self-report of medical diagnoses (yes / no)
	Diabetes mellitus	Self-reported diagnosis by a physician, self-reported intake of antidiabetic medication within the past two weeks, or measured blood glucose level of ≥ 126 mg/dl after an overnight fast of ≥ 8 h or a blood glucose level of > 200 mg/dl after a fasting period of ≥ 8 h
	Arterial hypertension	Measured systolic blood pressure ≥ 140 mm Hg or diastolic blood pressure ≥ 90 mm Hg at rest or self-reported intake of any antihypertensive drugs within the past 2 weeks or self-reported arterial hypertension diagnosed by a physician
	Dyslipidemia	LDL/HDL ratio greater than 3.5
	Obesity	Body mass index (BMI) > 30 kg / m^2^
	Alcohol consumption	Self-reported total amount, converted into pure alcohol in grams per day: cut-off point are 10 g/day for women and 20 g/day for men
	Smoking	Self-report (yes / no); if yes: number of years * number of packs per day
	Current physical condition	Self-rating, range from 1 = very good, 2 = good, 3 = less good to 4 = bad
*Genotyping and imputation of Single Nucleotide Polymorphisms*	KIBRA rs17070145 BDNF rs6265 DBH rs1611115 DRD1 rs4532 ANKK1 rs1800497 DRD2 rs6277 COMT rs4680 COMT rs4818 APOE rs429358 APOE rs7412	Affymetrix Genome-Wide Human SNP 6.0 array (Affymetrix, Santa Clara, CA)^44^. APOE SNPs (rs429358 and rs7412) were aggregated accordingly to include the epsilon 2, 3 and 4 variants in the analyses.

#### Statistical procedure

We first performed standard descriptive statistics for all variables and calculated Pearson correlations of TOL performance at baseline (T1) and follow-up (T2).

**Figure 1:**
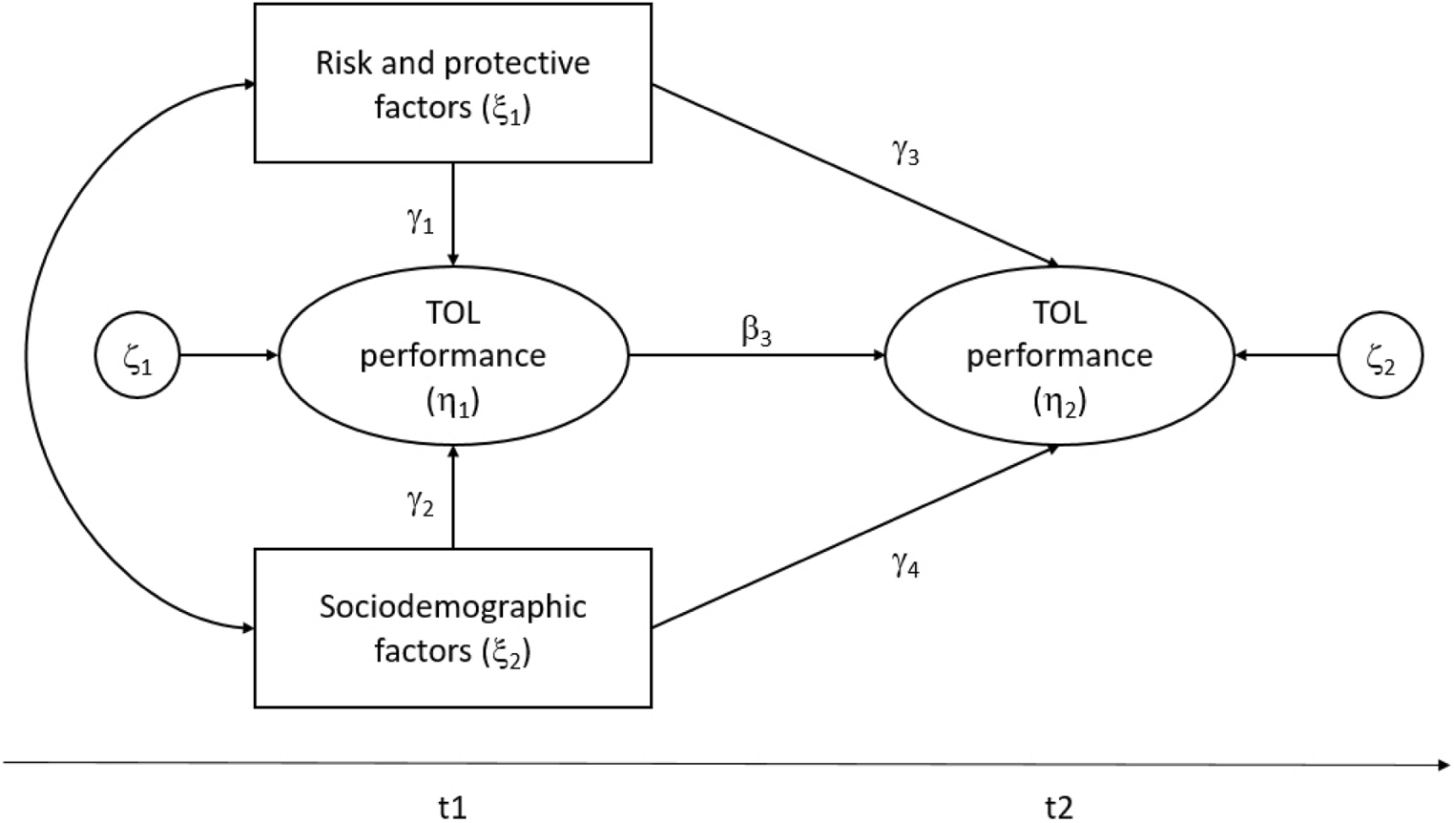
Auto-regressive model of the TOL performance.

We used structural equation models (SEM) to study the relations between risk and protective factors and TOL performance over time. Of particular interest for the present study was the relationship between risk, protective and sociodemographic factors at the first point in time on cognitive functioning at the second point in time while controlling for cognitive performance at the first time point (see Fig. 1). We investigated gender, education, living in a partnership, and social support as potential protective factors. As potential risk factors, we investigated genetic allele-variants (Table 2), medication as well as physical and mental health, and lifestyle factors as listed in Table 3.

**Table 2:** Overview of the SNPs of interest with the respective chromosomes, genes and the corresponding allele variants and the absolute number of observations.

Chromosome	SNP-ID	Gene	Wild-type	</≥ 60	Homozygote	</≥ 60	Heterozygote	</≥ 60
5	rs17070145	KIBRA	C	1352 / 578	T	250 / 144	CT	1162 / 532
11	rs6265	BDNF	G	1714 / 760	A	111 / 62	GA	939 / 432
9	rs1611115	DBH	T	79 / 34	C	1920 / 878	TC	765 / 342
5	rs4532	DRD1	C	350 / 171	T	1119 /496	CT	1295 / 587
11	rs1800497	ANKK1	C	1802 / 811	T	93 / 45	CT	869 / 398
11	rs6277	DRD2	C	545 / 246	T	820 / 383	CT	1399 / 625
22	rs4680	COMT	G	661 / 296	A	707 / 328	GA	1396 / 630
22	rs4818	COMT	C	998 / 448	G	433 / 188	CG	1333 / 618
19	rs429358	APOE	T	2103 / 977	C	52 / 18	TC	609 / 259
19	rs7412	APOE	C	2370 / 1069	T	16 / 11	CT	378 / 174

**Table 3:** Sample characteristics categorized into protective and risk factors.

	Sample	< 60 years	≥ 60 years	*p*
	*N* = 4,018	*N* = 2,764 (68.8%)	*N* = 1,254 (31.2%)	
***Protective Factors***				
* Sociodemographic characteristics /*				
Sex (male %)	2,128 (53.0%)	1,419 (51.3%)	709 (56.5%)	**0.002**
Partnership (%)				
with partner	3,060 (76.2%)	2,053 (74.3%)	1,007 (80.3%)	**0.000**
without partner	958 (23.8%)	711 (25.7%)	247 (19.7%)	**0.000**
Education in years (mean)	14.51 (2.33)	14.73 (2.28)	14.04 (2.37)	**0.000**
Social Support (mean)	20.29 (3.51)	20.3 (3.47)	20.27 (3.60)	0.779
***Risk Factors***				
* Medication*				
Antidepressant medication (yes %)	194 (4.8%)	133 (4.8%)	61 (4.9%)	1.000
Anxiolytic medication (yes %)	27 (0.7%)	16 (0.6%)	11 (0.9%)	0.387
Antihypertensive medication (yes %)	1,143 (28.4%)	560 (20.3%)	583 (46.5%)	**0.000**
Antidiabetic medication (yes %)	179 (4.5%)	72 (2.6%)	107 (8.5%)	**0.000**
* Physical, mental health and lifestyle factors*				
PHQ-9 (mean)	4.36 (3.57)	4.62 (3.67)	3.79 (3.27)	**0.000**
GAD-2 (mean)	0.99 (1.10)	1.06 (1.13)	0.83 (1.03)	**0.000**
Panic (yes %)	216 (5.4%)	160 (5.8%)	56 (4.5%)	0.099
Loneliness (yes %)	730 (18.2%)	520 (18.8%)	210 (16.7%)	0.126
Subjective physical health (mean)	2.07 (0.56)	2.07 (0.55)	2.08 (0.59)	0.615
Subjective mental health (mean)	2.03 (0.63)	2.04 (0.64)	1.99 (0.63)	**0.008**
Smoking (packs per day, mean)	0.10 (0.29)	0.12 (0.32)	0.06 (0.23)	**0.000**
Alcohol consumption (%)*	1,007 (25.1%)	612 (22.1%)	395 (31.5%)	**0.000**
Diabetes mellitus (yes %)	274 (6.8%)	118 (4.3%)	156 (12.4%)	**0.000**
Obesity (yes %)	957 (23.8%)	614 (22.2%)	343 (27.4%)	**0.000**
Hypertension (%)				
no	2,190 (54.5%)	1,738 (62.9%)	452 (36.1%)	**0.000**
known	1,351 (33.6%)	706 (25.5%)	645 (51.4%)	**0.000**
unknown	477 (11.9%)	320 (11.6%)	157 (12.5%)	0.422
Dyslipidemia (yes %)	1,215 (30.2%)	711 (25.7%)	504 (40.2%)	**0.000**
CVD (yes %)	229 (5.7%)	113 (4.1%)	116 (9.2%)	**0.000**
OPD (yes %)**	162 (4.0%)	107 (3.9%)	55 (4.4%)	0.495
Chronic liver disease (yes %)	15 (0.4%)	7 (0.3%)	8 (0.6%)	0.116
Chronic kidney disease (yes %)	29 (0.7%)	19 (0.7%)	10 (0.8%)	0.857
Cancer (yes %)	336 (8.4%)	154 (5.6%)	182 (14.5%)	**0.000**
Infection last week (yes %)	714 (17.8%)	519 (18.8%)	195 (15.6%)	**0.045**
Autoimmune disease (yes %)	277 (6.9%)	198 (7.2%)	79 (6.3%)	**0.020**

*p* *p*-value, significant p-values are printed in bold.

* > recommended limits of 10 g/day for women and 20 g/day for men.

** Obstructive pulmonary disease (COPD or asthma).

Given that our focus is not on hypothesis testing, but on exploring the diversity of potential factors that may influence cognitive change, no correction for multiple comparisons was applied. More detail on statistical modelling is provided in the Supplemental information under “Statistical Procedure“.

## Results

The descriptive data of the sample characteristics are presented in Table 3. Due to the increased morbidity in later life, as expected, several medication and disease parameters differ significantly between the younger and older group.

**Figure 2:**
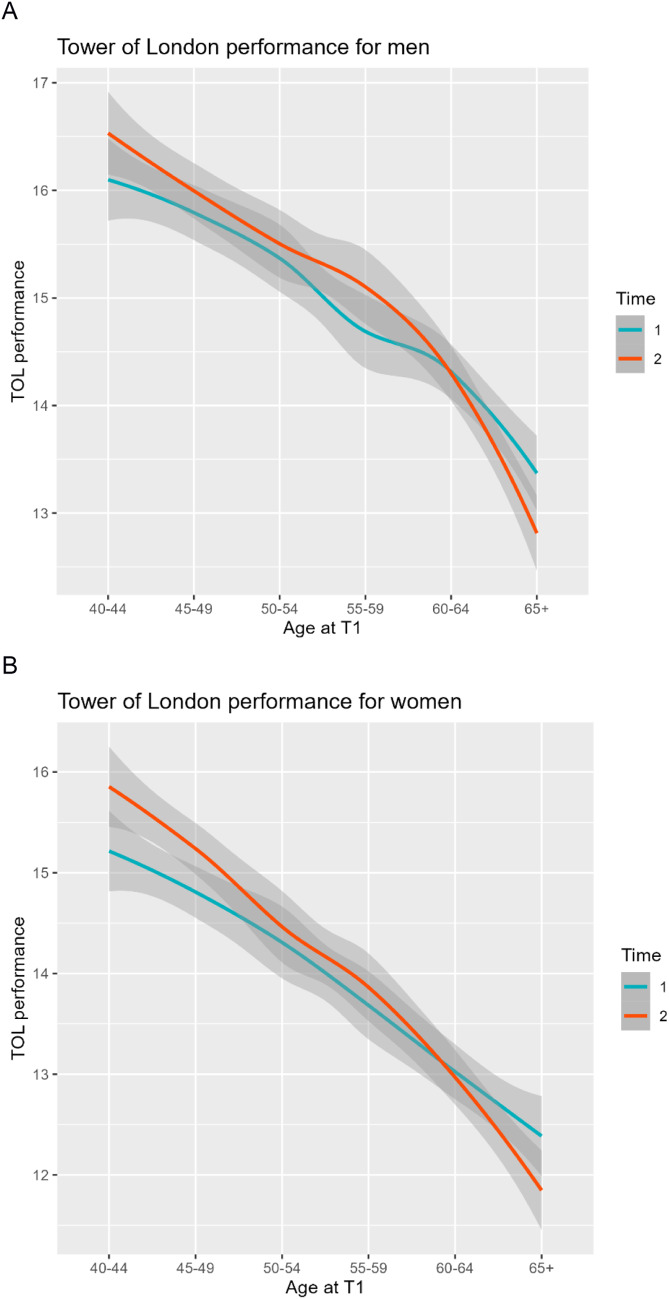
Average planning performance of the corresponding age groups for women (**A**) and men (**B**) at T1 (blue line) and T2 (red line).

As seen in Table 4; Fig. 2A and B, planning performance nominally increased from the first to the second measurement in each age group up to participants aged 55 to 59. From age 60 onwards, on the other hand, a reduction in performance over the five years between the two measurements was observed. A two-way repeated measures ANOVA of planning ability with the factors age group (< 60 / ≥ 60 years; between-subjects factor) and time (T1 and T2; within-subjects factor) was performed. The analysis revealed a significant main effect for age group (*F*(1,4016) = 334.36; *p* < .001; *partial η²* = 0.059) with better planning performance in the younger group, but not for time (*F*(1, 4016) = 0.010; *p* = .922; *partial η2* = 0.000), and a significant interaction of these factors (*F*(1, 4016) = 28.86; *p* < .001; *partial η2* = 0.002). The interaction indicated the inverted change of planning in the younger (increased performance from T1 *mean* = 14.95, *SD* = 3.38, to T2 *mean* = 15.27, *SD* = 3.35, respectively) as opposed to the older group (decreased performance from T1 *mean* = 13.41, *SD* = 3.46 to T2 *mean* = 13.11, *SD* = 3.61, respectively).

**Table 4:** Average planning performance of the respective age groups in the 1st and 2nd measurement for the overall sample and separated by gender.

	Full Sample		Men		Women	
Age groups at T1	COR at T1	COR at T2	COR at T1	COR at T2	COR at T1	COR at T2
40–44	15.69 (3.17)	16.20 (3.18)	16.11 (3.22)	16.53 (3.26)	15.22 (3.05)	15.83 (3.07)
45–49	15.24 (3.27)	15.63 (3.23)	15.75 (3.36)	16.01 (3.38)	14.80 (3.13)	15.31 (3.07)
50–54	14.91 (3.45)	15.06 (3.42)	15.37 (3.31)	15.50 (3.37)	14.31 (3.55)	14.46 (3.40)
55–59	14.18 (3.42)	14.48 (3.32)	14.69 (3.40)	15.10 (3.28)	13.68 (3.37)	13.86 (3.24)
60–64	13.86 (3.30)	13.74 (3.37)	14.52 (3.22)	14.36 (3.15)	13.01 (3.21)	12.95 (3.47)
65+*	12.91 (3.57)	12.39 (3.74)	13.31 (3.50)	12.79 (3.77)	12.39 (3.61)	11.85 (3.63)

* Comprises three participants older than 70 years. “COR” refers to the number of correctly solved tasks (maximum possible: 24). T1 and T2 refer to the 1st and 2nd measurement, respectively.

A Pearson correlation coefficient of *r* = .53 (*p* < .001) between T1 and T2 in the overall sample indicates a relatively stable measurement, which is a good basis for further predictions using structural equation modelling.

### Risk factors

Concerning genetic factors, significant results were obtained for both **COMT** variants in the overall model (rs4680: G/G vs. G/A: *β* = -0.37; *p* = .038; G/G vs. A/A: *β* = -0.50; *p* = .038; rs4818: G/G vs. C/G: *β* = 0.43; *p* = .037, C/C vs. G/G: *β* = -0.65; *p* = .010; see Table 5). These effects were also found in the younger age group. In the rs4680 COMT SNP, G/G allele carriers showed lower planning performance than G/A (*β* = -0.61; *p* = .004) and A/A individuals (*β* = -0.60; *p* = .033). These findings missed significance in the older group (*p* ≥ .05). However, the difference in the regression weights between both age groups for the G/G versus G/A contrast proved to be just below the critical significance threshold (Δ χ^*2*^ = 3.80; *p* = .051). For the rs4818 COMT SNP, carriers of the G/G variant revealed better performance than the C/G heterozygotes (*β* = 0.65; *p* = .005) and also than C/C carriers (C/C vs. G/G *β* = -0.84; *p* = .004) in the younger age group. Thus, both findings of the overall model were only present in the younger group.

**Table 5:** Results of the autoregressive models for the overall sample (model 1) and stratified by age (model 2). The Chi-Square Test checks for differences between the two age groups in model 2 (i.e. moderating effects of age).

	Model 1: Overall		Model 2: Multiple Group Analysis				Chi-Square Test	
			**Group 1: < 60**		**Group 2**: ≥ **60**			
	***N*** **= 4**,**018** **R**^**2**^ **= 0.333**		***N*** **= 2**,**764** **R**^**2**^ **= 0.317**		***N*** **= 1**,**254** **R**^**2**^ **= 0.238**			
	Estimate (SE)	p	Estimate (SE)	p	Estimate (SE)	p	Δ Chi	p
**SNPs**								
KIBRA								
C vs. CT	0.042 (0.098)	0.673	0.092 (0.113)	0.416	-0.089 (0.195)	0.648	0.629	0.428
T vs. CT	0.131 (0.169)	0.438	0.072 (0.200)	0.720	0.220 (0.311)	0.479	0.160	0.690
C vs. T	-0.090 (0.166)	0.588	0.020 (0.196)	0.918	-0.309 (0.305)	0.311	0.789	0.374
DRD1								
C vs. CT	-0.057 (0.146)	0.695	-0.030 (0.174)	0.864	-0.088 (0.269)	0.743	0.033	0.956
T vs. CT	-0.053 (0.099)	0.591	-0.096 (0.113)	0.395	0.013 (0.195)	0.946	0.236	0.627
C vs. T	-0.004 (0.148)	0.979	0.066 (0.174)	0.704	-0.101 (0.278)	0.716	0.259	0.611
DBH								
T vs. CT	-0.462 (0.319)	0.148	-0.441 (0.384)	0.250	-0.407 (0.547)	0.456	0.002	0.960
C vs. CT	-0.103 (0.103)	0.321	-0.141 (0.119)	0.239	-0.074 (0.202)	0.715	0.081	0.776
T vs. C	-0.360 (0.313)	0.250	-0.300 (0.376)	0.425	-0.334 (0.530)	0.529	0.003	0.959
DRD2 rs1800497								
C vs. CT	0.051 (0.104)	0.621	-0.107 (0.119)	0.368	0.441 (0.204)	**0.031**	4.841	**0.028**
T vs. CT	0.030 (0.272)	0.913	-0.210 (0.322)	0.514	0.605 (0.496)	0.223	1.914	0.166
C vs. T	0.022 (0.270)	0.936	0.103 (0.319)	0.746	-0.163 (0.491)	0.739	0.209	0.647
BDNF								
G vs. GA	-0.028 (0.099)	0.776	-0.104 (0.114)	0.360	0.164 (0.191)	0.391	1.508	0.219
A vs. GA	-0.103 (0.245)	0.674	-0.102 (0.299)	0.732	0.005 (0.414)	0.990	0.044	0.833
G vs. A	0.075 (0.239)	0.754	-0.002 (0.291)	0.994	0.159 (0.404)	0.695	0.104	0.747
DRD2 rs6277								
C vs. CT	-0.209 (0.129)	0.105	0.012 (0.142)	0.934	-0.638 (0.271)	**0.019**	4.361	**0.037**
T vs. CT	-0.152 (0.108)	0.160	0.059 (0.125)	0.636	-0.592 (0.207)	**0.004**	6.739	**0.009**
C vs. T	-0.057 (0.142)	0.687	-0.047 (0.157)	0.763	-0.046 (0.297)	0.878	0.000	0.996
COMT rs4680								
G (VAL) vs. GA	-0.373 (0.180)	**0.038**	-0.613 (0.211)	**0.004**	0.159 (0.329)	0.629	3.792	0.051
A (MET) vs. GA	0.123 (0.172)	0.475	-0.014 (0.203)	0.947	0.409 (0.330)	0.215	1.204	0.272
G vs. A	-0.496 (0.239)	**0.038**	-0.600 (0.281)	**0.033**	-0.250 (0.444)	0.573	0.437	0.508
COMT rs4818								
C vs. CG	-0.220 (0.161)	0.171	-0.183 (0.189)	0.331	-0.339 (0.306)	0.268	0.187	0.665
G vs. CG	0.427 (0.205)	**0.037**	0.653 (0.234)	**0.005**	-0.085 (0.397)	0.831	2.596	0.107
C vs. G	-0.647 (0.250)	**0.010**	-0.837 (0.289)	**0.004**	-0.254 (0.479)	0.595	1.091	0.296
APOE								
ε2ε2 and ε2ε3 vs. ε3ε3	-0.138 (0.134)	0.304	0.061 (0.152)	0.688	-0.602 (0.259)	**0.020**	5.709	**0.017**
ε4ε4 and ε4ε3 vs. ε3ε3	-0.224 (0.114)	**0.049**	-0.081 (0.129)	0.527	-0.534 (0.233)	**0.022**	2.983	0.084
ε4ε4 and ε4ε3 vs. ε2ε2 and ε2ε3	-0.086 (0.155)	0.578	-0.142 (0.175)	0.416	0.067 (0.316)	0.832	0.344	0.557
** COR**								
COR at T1	0.486 (0.015)	**0.000**	0.505 (0.018)	**0.000**	0.447 (0.030)	**0.000**	2.825	0.093
**Risk Factors**								
PHQ-9	0.024 (0.020)	0.212	0.023 (0.022)	0.306	0.014 (0.040)	0.733	0.038	0.844
GAD-2	-0.053 (0.063)	0.394	-0.105 (0.069)	0.129	0.110 (0.140)	0.431	1.836	0.175
Panic	0.354 (0.232)	0.127	0.385 (0.264)	0.145	0.340 (0.484)	0.483	0.007	0.934
Loneliness (binary)	-0.065 (0.139)	0.638	-0.088 (0.161)	0.583	0.072 (0.272)	0.792	0.257	0.612
Antidepressant medication	-0.202 (0.220)	0.359	0.085 (0.236)	0.717	-0.979 (0.462)	**0.034**	4.319	**0.038**
Antihypertensive medication	0.115 (0.222)	0.606	0.037 (0.265)	0.888	0.081 (0.396)	0.839	0.008	0.927
Antidiabetic medication	0.140 (0.400)	0.727	-0.159 (0.572)	0.780	0.476 (0.553)	0.389	0.655	0.418
Subjective physical health	0.024 (0.095)	0.799	0.046 (0.112)	0.683	0.064 (0.175)	0.716	0.007	0.932
Subjective mental health	-0.106 (0.096)	0.270	-0.067 (0.110)	0.544	-0.211 (0.187)	0.260	0.424	0.515
Smoking (packs per day)	-0.062 (0.166)	0.711	-0.230 (0.183)	0.208	0.595 (0.381)	0.118	3.809	0.051
Alcohol consumption (over the recommended limit)	-0.213 (0.109)	0.051	-0.038 (0.126)	0.765	-0.439 (0.204)	**0.032**	2.785	0.095
Diabetes	-0.023 (0.339)	0.947	0.227 (0.484)	0.639	-0.310 (0.473)	0.512	0.661	0.416
Obesity	-0.157 (0.118)	0.183	-0.204 (0.138)	0.138	-0.078 (0.221)	0.723	0.237	0.627
Hypertension (Ref. no Hypertension)								
known	-0.343 (0.210)	0.103	-0.402 (0.242)	0.098	-0.041 (0.407)	0.920	0.583	0.445
unknown	-0.364 (0.150)	**0.015**	-0.606 (0.174)	**0.000**	0.255 (0.290)	0.381	6.420	**0.011**
Dyslipidemia	0.025 (0.109)	0.817	0.084 (0.127)	0.507	-0.006 (0.198)	0.977	0.147	0.701
CVD	-0.167 (0.217)	0.441	-0.287 (0.292)	0.326	0.064 (0.318)	0.840	0.648	0.421
OPD	0.391 (0.244)	0.109	0.756 (0.299)	**0.012**	-0.276 (0.407)	0.497	4.247	**0.039**
Cancer	-0.109 (0.174)	0.529	-0.210 (0.215)	0.329	0.009 (0.262)	0.973	0.419	0.517
Infection last week	-0.001 (0.004)	0.900	0.003 (0.005)	0.521	-0.009 (0.008)	0.274	1.726	0.189
Autoimmune disease	-0.011 (0.004)	**0.005**	-0.005 (0.004)	0.238	-0.015 (0.007)	**0.035**	1.490	0.222
** Protective Factors**								
Social Support	0.005 (0.015)	0.755	-0.001 (0.018)	0.936	0.008 (0.027)	0.766	0.084	0.771
Sociodemographic Factors								
Sex (Male)	0.445 (0.100)	**0.000**	0.432 (0.113)	**0.000**	0.464 (0.195)	**0.017**	0.021	0.885
Age (≥ 60, Ref.: < 60)	-1.247 (0.114)	**0.000**	-	-	-	**-**		
Partnership (yes)	0.136 (0.113)	0.229	0.037 (0.128)	0.770	0.479 (0.237)	**0.043**	2.614	0.106
Education (years)	0.134 (0.021)	**0.000**	0.167 (0.025)	**0.000**	0.066 (0.039)	0.092	4.046	**0.044**

*Estimate* = unstandardized estimate, *SE* = standard error, *p* = *p*-value, significant *p*-values are printed in bold

Two D2 dopamine receptor (**DRD2**) SNPs predicted planning performance in the older age group only: Carriers of the C/C allele increased performance compared to heterozygotes (C/T) in the rs1800497 SNP (*β* = 0.44; *p* = .031). There was also a significant difference between the age groups (Δχ^*2*^ = 4.84; *p* = .028), indicating a moderating effect of age. For the rs6277 SNP, both C/C and T/T individuals had lower performance compared to heterozygotes (C/T) (*β* = -0.64; *p* = .019, and *β* = -0.59; *p* = .004, respectively), and for both SNPs there was a significant moderating effect of age (Δχ^*2*^ = 4.36; *p* = .037 and Δχ^*2*^ = 6.74; *p* = .009, respectively).

For the **APOE** gene, carriers of the ε4-variant reached lower TOL-performance than the ε3 allele in the overall sample (*β* = -0.22; *p* = .049) and the older group (*β* = -0.53; *p* = .022). Individuals with the ε2-variant also had lower performance than ε3 carriers, but only in the group of 60 years and older (*β* = -0.60; *p* = .020). This effect differed significantly between the two age groups (Δχ^*2*^ = 5.71; *p* = .017).

In addition to genetic factors, **disease-related and lifestyle risk** factors also affected cognitive changes. We found a significant negative association with antidepressant medication in the older group only (*β* = -0.98; *p* = .034). Thus, in individuals aged 60 years or older, performance decrease could be observed when they were pharmacologically treated for depression. This finding was moderated by age, as predictive power proved to be significantly lower in the younger group (Δχ^*2*^ = 4.32; *p* = .038).

Another effect in the older group concerned the amount of alcohol consumed. For people aged 60 or above, drinking more than the recommended amount of alcohol was associated with poorer planning performance (*β* = -0.44; *p* = .032).

A very interesting result also emerged with hypertension. If hypertension was not known and in consequence, untreated, this resulted in an overall negative prediction of cognitive performance five years later (*β* = -0.36; *p* = .015). However, this negative relationship was only valid in the younger age group (*β* = -0.61; *p* < .001), not at older age (*β* = 0.26; *p* = .381), resulting in a significant difference between the two age groups (Δχ^*2*^ = 6.42; *p* = .011).

With respect to chronic diseases, obstructive pulmonary disease (OPD; chronic OPD or asthma) was identified as a significant predictor in the younger participants (*β* = 0.76; *p* = .012). They revealed higher cognitive performance after five years, and the difference between the age groups attained significance (Δχ^*2*^ = 4.30; *p* = .039). Finally, the presence of an autoimmune disease had a negative predictive value on planning performance according to the analysis of the group aged 60 or older (*β* = -0.02; *p* = .035) and the overall sample (*β* = -0.01; *p* = .005).

### Protective factors

Regarding demographic factors, male sex positively predicted better planning performance after five years (*β* = 0.45; *p* < .001). This effect was found both in the younger and the older age groups (*β* = 0.43; *p* < .001, and *β* = 0.46; *p* = .017, respectively).

In addition, education was shown to be protective, as more highly educated participants showed better planning performance in the second measurement (*β* = 0.13; *p* < .001). This effect was more pronounced in the younger group (*β* = 0.17; *p* < .001), resulting in a significant difference between the two age groups (Δχ^*2*^ = 4.05; *p* = .044). Living in a partnership only resulted in better planning ability in the older group (β = 0.48; *p* = .043).

## Discussion

The behavioral results of this study are consistent with previous longitudinal observations that participants from the seventh decade of life show a decrease in performance for the second measurement instead of a re-test benefit in younger persons. Thus, our behavioral data provide an ideal starting point to identify genetic, disease-related, lifestyle and sociodemographic factors that relate to retained vs. declined performance dependent on age group.

Age-stratified models showed that the predictive value of some genetic factors affecting the dopamine system varied with age, extending previous research. Below 60 years, carriers of the G/G (or Val/Val) allele of the rs4680 COMT SNP showed decreased planning performance compared to G/A (Val/Met) and A/A (Met/Met) individuals. This is in line with the assumption that adult Met/Met carriers are closer to the optimum level of dopamine, reflected in better cognitive performance than Val/Val carriers^34^. Tsuchimine et al.^45^, on the other hand, reported better TOL performance in Val/Val individuals in their sample of young adult participants, which, however, may be due to age or ethnicity-related differences between Asian and Caucasian samples^46^. Another SNP, rs4818, was suggested to substantially contribute to dopaminergic variation based on a small sample of young male adults^47^; (*N* = 107 males; mean age 25.5 years). In this sample, C/C individuals performed best, G/G worst, and C/G intermediate in a planning task. In our study, carriers of the G/G variant revealed better performance than the C/G heterozygotes and C/C carriers in the younger age group (< 60) at T2. Again, these variations can also be due to the young and cross-sectional sample of Roussos et al.^47^.

For the rs6277 SNP on the DRD2 gene, both C/C and T/T individuals had lower performance than heterozygotes in the older group. This is partly in line with^48^ studying complex planning in 122 healthy adult males (mean age 35.2 years) without consideration of aging. They also showed that C/C homozygotes solved fewer problems compared to C/T heterozygotes, while T/T homozygotes’ scores lay in-between. The DRD2 TaqIA polymorphism (rs1800497) regulates density of D2 receptors in the striatum, with highest density in allele C/C^49,50^. Associations to cognition are heterogenous^51^, even to executive functions^52^. In our study, carriers of the C/C allele showed increased performance compared to heterozygotes (C/T). Notably, all DRD2-related effects also significantly differed between age groups, underlining the importance of the fronto-striatal loop for executive functions in older age.

APOE is a major predictor of cognitive decline in people with and without dementia^53^. In addition to brain-related effects, APOE may display indirect effects on cognition via hemodynamic / vascular factors such as vasoreactivity (e.g^54^). It has become widely accepted that the ε4 allele is associated with a higher risk of Alzheimer’s disease (AD), while the ε2 allele lowers risk of AD^55^. Lower planning performance after five years for the ε4-variant in the older age group and the overall sample accord with this notion. Contrary to expectations, however, in the older age group, the ε2-variant also revealed lower planning performance than ε3, suggesting that ε2’s positive effects may not comprise complex executive functions. To summarize, COMT SNPs primarily predicted cognitive changes in younger age, and the DRD2 SNPs and APOE ε variants in older age. Results thus conform only partly with the phenomenon termed “aging-related magnification”^26^. One explanation for the lack of the aging-related magnification effect in some candidate SNPs could be the age distribution of the present sample starting at age 40. Other studies have used an extreme groups approach comparing young adults at the age of around 20 with older participants over 60 to 80 years, or covered the whole age range. Also, in contrast to many other studies on executive functions, we did not compare performance in middle-aged versus older adult participants, but targeted the 5-year-change of planning performance. Even though genetic predisposition cannot be changed, in the future, knowledge of genetic risk factors may nonetheless be used to screen and inform patients, and to possibly underline the need to improve modifiable factors such as lifestyle.

Looking at disease-related and lifestyle factors, we found that antidepressant medication, unknown hypertension, and OPD showed significantly age-moderated predictive values for planning performance. Significant detrimental effects of alcohol consumption and the prevalence of autoimmune disease were only observed within the older age group.

Among older participants, antidepressant medication, but not depression itself, predicted reduced performance. Antidepressants’ effects on cognition at an older age are controversial^56^. Leng et al.^57^ reported an increased risk of cognitive impairment among their oldest group of females which they attribute to potentially detrimental anticholinergic properties of many antidepressants^58,59^.

With respect to alcohol consumption, drinking more than the recommended amount of alcohol was associated with poorer planning performance for people aged 60 or older. This negative association was significantly stronger than in the younger group, indicating its particular importance of older age. Based on recent studies, moderate consumption is believed to exert a protective influence on mental health, whereas abstinence and heavy drinking negatively influence cognitive performance^60,61^. However, there is considerable variation in the definition of moderate alcohol consumption between studies, ranging from “once or twice a month”^60^, to “10 to 14 drinks per week”^61^, or “14 to 21 units per week” in the Whitehall II study^62^. Despite limited comparability, findings indicate an adverse effect of high alcohol consumption on cognitive performance at older age.

Hypertension has often been described as a risk factor for cognitive decline and dementia^53^. Especially mid-life-hypertension^2^ strongly relates to cognitive decline, as early hypertension most likely means longer risk exposure. Some studies even report protective effects of late-life hypertension against cognitive decline^63,64^ possibly through maintenance of perfusion. We did not find any effects of diagnosed hypertension. But undiagnosed and untreated hypertension was associated to decline of cognitive performance. This relationship was strongly expressed in the younger group and differed significantly from the older group. Consequently, diagnosis and treatment of hypertension in midlife has been confirmed as an important measure to prevent cognitive impairment at an early stage.

Finally, two chronic diseases showed significant associations with changes in planning performance in our analyses: obstructive pulmonary disease (COPD or asthma) and the presence of an autoimmune disease. COPD can be associated with cognitive impairment due to tissue hypoxemia and other mechanisms like cerebrovascular regulation disorder and systemic inflammation^65^. However, cognitive deficits were not consistently observed; these were most pronounced in severe asthma^66^. In our study, obstructive pulmonary disease in the younger group predicted relatively better planning performance five years later, which is inconsistent with the previous literature. However, as COPD, asthma, their severity and progression were not examined differentially, we could not determine the reasons for this unexpected finding. As all participants obtained a health record including pulmonary functions at each assessment, this may have contributed to more effective treatment and smoking cessation in participants with OPDs.

The presence of an autoimmune disease had a negative effect on planning performance according to the analysis of the overall sample and the group aged 60 or older. Although statistically significant, the number of affected participants is small. Since there are many different forms of autoimmune diseases and we did not categorize them more precisely in this study, in-depth data interpretation is impossible.

The effects of disease and medication on changes in cognition are important issues that should be taken into account by public healthcare policy, general practitioners and internal medicine physicians and communicated to patients. The same applies to lifestyle factors such as alcohol consumption.

When turning to protective factors, male sex, longer education, and living in a partnership were associated with better planning performance after five years. The effects of education and sex on planning concur well with the cross-sectional findings of D’Antuono^67^ and Boccia et al.^68^ and replicate the results of Kaller et al.^69^. Sex effects were ascribed to the visual-spatial layout of the TOL and trajectories of steroid hormone levels over the male versus female adult life span^70^.

Higher education is associated with better health through enhanced resources that enable a healthier lifestyle, resulting in better brain health and cognitive performance^71^. Our findings expand on previous cross-sectional investigations of planning^14^ by showing that male sex and higher education exert protective influences over time as well. Whereas male sex was strongly predictive of changes in planning performance in both age groups, education attained statistical significance in the younger group only. In older, usually no longer occupationally active participants, the importance of education may decrease compared to other variables, such as physical and cognitive activity.

Consistent with previous findings, living in a relationship has a protective effect against cognitive impairment later in life^72^. This may be due to advantageous financial resources, better health behavior, increased cognitive stimulation, and higher levels of social integration^73^.

In particular, our findings regarding education and living together is consistent with activities commonly assumed to positively impact cognition such as cognitive challenges, acquiring new knowledge, but also cognitive stimulation from social interaction, still play a significant role in old age. This evidence should also be communicated by public health representants and senior citizens’ associations and focused on in old people’s homes.

### Limitations

Important parameters such as physical activity or hearing loss could not be included due to incomplete data. In addition, the sample size differed between age groups, limiting statistical power. Formation of two broad age groups likewise limited power by leaving age-related within-group differences unmodeled. Due to the exploratory character of the study, no correction for multiple comparisons was applied, requiring independent replication of the reported results. Furthermore, we could not differentiate between COPD and asthma or grade their severity, so that the unexpected positive association to planning could not be resolved. The negative prediction of APOE ε2 on cognitive change was inconsistent with the literature. Further data collection and more in-depth analyses, including interactions between protective and risk factors, are needed.

Study participation required on-site visits. Older adults living in nursing homes or who moved there, were probably underrepresented, resulting in selection bias and drop-out of particularly vulnerable individuals. However, the population-representative sampling is a great strength of the study. Identifying differential trajectories allowed for a combined exploration of potential risk/protective factors representing diverse life domains and influences.

## Conclusion

Stratification by age revealed genetic, lifestyle and health factors that predict 5-year change in cognitive performance. Most notably, differential predictors were found in younger vs. older participants. In consequence, retaining cognitive performance into old age by enhancing health and lifestyle should start in early midlife. These findings should be incorporated into preventive programs and health policy decisions, and should also be used to inform patients by general practitioners.

## Supplementary information

Below is the link to the electronic supplementary material.

Supplementary Material 1 (DOCX 62 KB)

## Data Availability

The datasets analysed during the current study are not publicly available. The written informed consent of the study participants is not suitable for public access of the data and this concept was not approved by the local data protection officer and ethics committee. But access to data at the local database in accordance with the ethics vote is offered upon reasonable request at any time. Interested researchers make their requests of the Principal Investigator of the GHS (Philipp.Wild@unimedizin-mainz.de).
