# Supplementary material for: Different risk and protective factors predict change of planning ability in middle versus older age
Source: Sci Rep. 2024 Oct 25;14:25275. doi: 10.1038/s41598-024-76784-1 (PMC11511955; doi:10.1038/s41598-024-76784-1)
Supplement: Supplementary file 1 — Supplementary Material 1 (DOCX 62 KB) [file 41598_2024_76784_MOESM1_ESM.docx]

**Supplementary information**

***Data collection and assessments***

*Planning ability: Tower of London –* *Freiburg Version (TOL-F)*

The TOL-F (1) is a psychometrically validated computerized version of the Tower of London implemented in the Vienna Test System (VTS; https://www.schuhfried.com/test/TOL-F https://marketplace.schuhfried.com/en/TOL).

In the TOL-F, individual problems consist of a start and a goal state presented in the computer screen's lower and upper halves, respectively. Subjects are instructed to transform the start into the goal state in the indicated minimum number of moves. Written instructions inform the participant about task operation and rules. Instructions further emphasize that participants should always plan ahead the problem solution before starting with movement execution.

The TOL-F is operated by touch screen. During instruction, participants’ task comprehension was controlled by two 2-move problems. After an additional set of four 3-move problems to get used to the task procedure, the proper testing started. It comprised 4-, 5-, and 6-move problems (eight of each) presented in increasing minimum number of moves. The instruction and practice phase were scheduled to take 5 min. A time limit of 20 min was applied for the testing of the 24 problems.

For assessment of individual planning ability, overall planning accuracy, defined as the percentage of problems that were correctly solved in the minimum number of moves, is the outcome variable of interest.

The TOL-F was the only cognitive test during the GHS procedure. It was embedded in a series of non-cognitive medical examinations comprised in the GHS.

*Socioeconomic factors and social support*

Sociodemographic data comprised participants’ sex/gender, age, and partnership (categorized as yes / no), and level of education (in years).

*Social support* was measured using the reliable Brief Social Support Scale (BS-6; (2)).

*Mental distress measures*

*Loneliness* was assessed using a validated single item (3,4). Participants responded to the question “I am frequently alone/have few contacts” on a five-point Likert scale.

*Anxiety symptoms* were captured using the GAD-2 using a validated cut-off of ≥ 3 for the overall sum score which ranges from 0 to 6 (5).

*Depression symptoms* were measured using the Patient Health Questionnaire’s depression module. Clinically relevant symptoms have been defined by a cut-off-score of ≥10 (total range: 0 to 27) (6).

*Panic disorder* was screened with the brief PHQ panic module. Caseness was assumed if at least two of the first four PHQ panic questions are answered with “yes” (7).

Patients were also asked to report on their current antidepressant and/or anxiolytic medication and rated their current mental state (ranging from 1 = very good, 2 = good, 3 = less good to 4 = bad).

*Physical health and health behavior*

*Cancer, cardiovascular disease, obstructive pulmonary disease (chronic OPD (COPD) and/or asthma), autoimmune diseases, chronic liver disease, chronic kidney disease, and infection last week* were assessed via self-reported medical diagnoses.

*Diabetes* mellitus was defined as a definite diagnosis of diabetes by a physician, as the intake of antidiabetic medication within the past two weeks, or as a blood glucose level of ≥126 mg/dl after an overnight fast of ≥8 hours or a blood glucose level of >200 mg/dl after a fasting period of ≥8 hours.

Arterial hypertension was defined as systolic blood pressure ≥140 mm Hg or diastolic blood pressure ≥90 mm Hg at rest or by intake of any antihypertensive drugs within the past 2 weeks or arterial hypertension diagnosed by a physician. Participants’ medication were categorized according to the Anatomical Therapeutic Chemical Classification System.

*Dyslipidemia* was identified with an LDL/HDL ratio greater than 3.5.

*Obesity* was defined as a Body mass index (BMI) >30 kg / m². Participants’ height was measured and they were weighed.

Participants’ *alcohol consumption* was assessed via self-report of how often, how many, and which kinds of beverages they consumed. Following a standardized procedure, the total amount of pure alcohol in grams per day was calculated. The cut-off point reflects the recommended limits in Germany which are 10 grams/day for women and 20 grams/day for men.

Participants were also asked whether they *smoked* and if yes, how many cigarettes/packs per day.

Years of smoking were calculated as the number of cigarettes smoked per day divided by 20 (a pack) and multiplied by the number of years smoked.

Participants were asked to rate their current physical condition (ranging from 1 = very good, 2 = good, 3 = less good to 4 = bad).

*Blood sampling and laboratory measurements*

Venous blood sampling was performed using tubes containing K3-ethylenediaminetetraacetic acid (EDTA). Platelet parameters were determined on an Advia 120 Hematology System (Siemens, Germany). Biochemical analysis were determined within 1 hour after sampling by routine methods in the central laboratory of University Medical Center Mainz.

*Genotyping and imputation of Single Nucleotide Polymorphisms (SNPs)*

We selected 10 SNPs (see Table 1) previously associated with cognitive functioning and decline (8,9). The two APOE SNPs (rs429358 and rs7412) were aggregated accordingly to include the Epsilon 2, 3 and 4 variants in the model analyses. (ε2ε2 or ε2ε3: *N* = 394 < 60, 185 ≥60; ε3ε3: *N* = 1,709 < 60, 792 ≥ 60; ε4ε4 or ε4ε3: *N* = 661 < 60, 277 ≥ 60). Genotyping was conducted on the Affymetrix Genome-Wide Human SNP 6.0 array (Affymetrix, Santa Clara, CA) according to the manufacturer´s recommendations (for further details, see (10)).

***Statistical Procedure***

We first performed standard descriptive statistics for all variables and calculated Pearson correlations of TOL performance at baseline (T1) and follow-up (T2).

We used structural equation models (SEM) to study the relations between risk and protective factors and TOL performance over time. A major approach for the analysis of panel data is the autoregressive model (11–14). The autoregressive model specifies and estimates stability coefficients (TOL performance T1), which reflect the amount of change in the relative rank order of individuals between two or more points in time (11). Hence, importantly, the stability coefficients do not bear information about the individual change in absolute scores. In our case, the cross-lagged autoregressive model is especially suited for the analyses of the two-wave panel data (14–16). Of particular interest for the present study was the relationship between risk, protective and sociodemographic factors at the first point in time on cognitive functioning at the second point in time while controlling for cognitive performance at the first time point (see Fig. 1). We investigated gender, education, living in a partnership, and social support as potential protective factors. As potential risk factors, we investigated genetic allele-variants (Table 2), medication as well as physical and mental health, and lifestyle factors as listed in Table 3.

This is expressed in the following equations:

η_1_ = γ_1_ξ_1_ + γ_2_ξ_2_ + ζ_1_  (1)

*Note. γ_1_,γ_2_ = partialized unstandardized regression coefficients of risk and protective factors as well as sociodemographic factors; η_1_ = TOL performance at T1; ξ_1_ = risk and protective factors; ξ_2_ = sociodemographic variables; ζ_1_ = error term. To simplify the equation, we have summarized here risk and protective factors under ξ_1_ and sociodemographic factors under ξ_2_. However, in the empirical test we tested the effects of all mentioned variables. The equation for the second time point is as follows:*

η_2_ = βη_1_ + γ_3_ξ_1_ + γ_4_ξ_2_ + ζ_2_ (2)

*Note. β = partialized unstandardized regression coefficient which represents the respective effect size of TOL performance at T1 on TOL performance at T2 (also called stability coefficient); γ_3_, γ_4_ = partialized unstandardized regression coefficients of risk and protective factors as well as sociodemographic factors; η_1_ = TOL performance at T1; η_2_ = TOL performance at T2; ξ_1_ = risk and protective factors at T1; ξ_2_ = sociodemographic variables at T1; ζ_2_ = error term.*

Equation 2 implies that predictors and control variables directly affect cognitive functioning at the second time point. As the model was just identified (df = 0), no global fit measures like χ^2^, RSMEA, SRMR, CFI, or TLI were supplied (17). To test for moderation effects, we applied a multi-group SEM. The group-specific regression weights are freely estimated in a first model test. In a second model estimation, the regression weights for both comparison groups (under 60 years vs. 60 years and older) are restricted to be equal. Since the latter is a nested model, the difference between the two χ^2^-values can be used to test whether parameter equating between the groups leads to significantly inferior data modeling. Significance implies that group-specific regression weights have to be assumed or that a significant inequality between the groups applies. A significant Δχ^2^-value thus implies that a moderation effect of age exists. All statistical analyses were performed using R Studio (version 1.3.1093), primarily the lavaan-package (18). Given that our focus is not on hypothesis testing, but on exploring the diversity of potential factors that may influence cognitive change, no correction for multiple testing was applied.

Note that for statistical analyses, only parameters with frequencies greater than or equal to 1% were included in the disease categories, as the other, infrequent characteristics are not representative and unnecessarily increase the number of degrees of freedom.

**References**

1. Kaller CP, Unterrainer JM, Kaiser S, Weisbrod M, Aschenbrenner S. Tower of London - Freiburger Version. Mödling: Schuhfried; 2012.

2. Beutel ME, Brähler E, Wiltink J, Michal M, Klein EM, Jünger C, et al. Emotional and tangible social support in a German population-based sample: Development and validation of the Brief Social Support Scale (BS6). Kocalevent RD, editor. PLOS ONE. 2017 Oct 12;12(10):e0186516.

3. Beutel ME, Klein EM, Brähler E, Reiner I, Jünger C, Michal M, et al. Loneliness in the general population: prevalence, determinants and relations to mental health. BMC Psychiatry. 2017 Dec;17(1):97.

4. Reinwarth AC, Ernst M, Krakau L, Brähler E, Beutel ME. Screening for loneliness in representative population samples: Validation of a single-item measure. Yon DK, editor. PLOS ONE. 2023 Mar 16;18(3):e0279701.

5. Löwe B, Wahl I, Rose M, Spitzer C, Glaesmer H, Wingenfeld K, et al. A 4-item measure of depression and anxiety: Validation and standardization of the Patient Health Questionnaire-4 (PHQ-4) in the general population. J Affect Disord. 2010 Apr;122(1–2):86–95.

6. Kocalevent RD, Hinz A, Brähler E. Standardization of the depression screener Patient Health Questionnaire (PHQ-9) in the general population. Gen Hosp Psychiatry. 2013 Sep;35(5):551–5.

7. Löwe B, Gräfe K, Zipfel S, Spitzer RL, Herrmann-Lingen C, Witte S, et al. Detecting panic disorder in medical and psychosomatic outpatients. J Psychosom Res. 2003 Dec;55(6):515–9.

8. Papenberg G, Lindenberger U, Bäckman L. Aging-related magnification of genetic effects on cognitive and brain integrity. Trends Cogn Sci. 2015 Sep;19(9):506–14.

9. Papenberg G, Salami A, Persson J, Lindenberger U, Bäckman L. Genetics and Functional Imaging: Effects of APOE, BDNF, COMT, and KIBRA in Aging. Neuropsychol Rev. 2015 Mar;25(1):47–62.

10. Panova-Noeva M, Arnold N, Hermanns MI, Prochaska JH, Schulz A, Spronk HM, et al. Mean Platelet Volume and Arterial Stiffness – Clinical Relationship and Common Genetic Variability. Sci Rep. 2017 Jan 6;7(1):40229.

11. Finkel S. Causal Analysis with Panel Data [Internet]. 2455 Teller Road, Thousand Oaks California 91320 United States of America: SAGE Publications, Inc.; 1995 [cited 2022 May 25]. Available from: https://methods.sagepub.com/book/causal-analysis-with-panel-data

12. Hertzog C, Nesselroade JR. Assessing Psychological Change in Adulthood: An Overview of Methodological Issues. Psychol Aging. 2003 Dec;18(4):639–57.

13. Jöreskog KG. Statistical estimation of structural models in longitudinal development investigations. In: Nesselroade JR, Baltes PB, editors. Longitudinal research in the study of behavior and development. New York: Academic Press; 1979. p. 303–52.

14. Little TD. Longitudinal structural equation modeling. New York: The Guilford Press; 2013. 386 p. (Methodology in the social sciences).

15. Kenny DA. Cross-lagged panel correlation: A test for spuriousness. Psychol Bull. 1975 Nov;82(6):887–903.

16. Kuiper RM, Ryan O. Drawing Conclusions from Cross-Lagged Relationships: Re-Considering the Role of the Time-Interval. Struct Equ Model Multidiscip J. 2018 Sep 3;25(5):809–23.

17. Kline RB. Principles and Practice of Structural Equation Modelling. 4th ed. London: The Guilford Press; 2016.

18. Rosseel Y. **lavaan** : An *R* Package for Structural Equation Modeling. J Stat Softw [Internet]. 2012 [cited 2023 Oct 30];48(2). Available from: http://www.jstatsoft.org/v48/i02/
